# Impacts of Different Functional Groups on the Kinetic Rates of α-Amine Ketoximesilanes Hydrolysis in the Preparation of Room Temperature Vulcanized Silicone Rubber

**DOI:** 10.3390/ma11050790

**Published:** 2018-05-13

**Authors:** Huihui Xu, Zihou Liu, Qingyang Liu, Yiling Bei, Qingzeng Zhu

**Affiliations:** 1 Key Laboratory of Special Functional Aggregated Materials, Ministry of Education, School of Chemistry and Chemical Engineering, Shandong University, Jinan 250100, China; 201411546@mail.sdu.edu.cn (H.X.); EBLX22@163.com (Z.L.); qzzhu@sdu.edu.cn (Q.Z.); 2 College of Biology and the Environment, Nanjing Forestry University, Nanjing 210037, China; liuqingyang0807@aliyun.com

**Keywords:** α-amine ketoximesilanes, hydrolysis reaction, self-crosslinkers, RTV silicone rubber

## Abstract

α-Amine ketoximesilanes are proven to be effective crosslinkers in the preparation of ketone-oxime one-component room temperature vulcanized (RTV) silicone rubber without the use of toxic metal catalyst. This work aimed to investigate the hydrolysis kinetic of α-amine ketoximesilanes, which is vitally important for the preparation of RTV silicone rubber. Five kinds of α-amine ketoximesilanes, namely α-(N,N-diethyl)aminomethyltri(methylethylketoxime)silane (DEMOS), α-(N,N-di-n-butyl)aminomethyltri(methylethylketoxime)silane (DBMOS), α-(N-n-butyl)aminomethyltri(methylethylketoxime)silane (n-BMOS), α-(N-cyclohexyl)aminomethyltri(methylethylketoxime)silane (CMOS) and α-(β-aminomethyl)aminomethyltri(methylethylketoxime)silane (AEMOS), were successfully obtained and confirmed using Fourier transform infrared spectrometer (FT-IR) and hydrogen-1 nuclear magnetic resonance ( ^1^H NMR). Kinetics of hydrolysis reactions were measured by FT-IR and conductivity. Our results illustrated that the kinetic constant rates ranged from 12.2 × 10^−4^ s^−1^ to 7.6 × 10^−4^ s^−1^, with the decreasing order of DEMOS > n-BMOS > DBMOS > CMOS > AEMOS at the given temperature and humidity. Better performances of thermal stability could be achieved when using the α-amine ketoximesilanes as crosslinkers in the preparation of RTV silicon rubber than that of RTV silicone rubber with the use of methyltri(methylethylketoxime)silane (MOS) as a crosslinker and organic tin as a catalyst.

## 1. Introduction

Silicone rubber that can vulcanize at room temperature is called room temperature vulcanized (RTV) silicone rubber. RTV silicone rubber is a kind of elastomer comprising silicone rubber gum (linear polysiloxane, PDMS), crosslinkers (methyltriethoxysilane), catalysts (dibutyltindilaurate), fillers (e.g., amorphoussilica), and other additives including antistatic agent [1,2]. Compared with traditional organic rubber, RTV silicone rubber has good temperature tolerance, good chemical resistance, good permeability, as well as excellent electrical-insulating properties [3,4,5]. For example, silicone rubber consisting of PDMS and graphene oxide is not only used for high-voltage insulations, but can also be used for high voltage field grading materials [6]. Thus, it has been widely used in aviation industry, electrical and electronic equipment, medical treatment and air purification [7,8,9].

Usually, commercial RTV silicon rubber, which is cross-linked using methyltriethoxysilane, has low mechanical and thermal properties because of weak Si-O-Si inter-molecular interactions in RTV silicone rubber [10]. Many studies have been launched to improve their properties including changing silicone rubber gum molecular structure, increasing gum molecular weight, using hybrid gum, mixing reinforcing fillers, vulcanizing auxiliaries as well as using different crosslinkers or catalysts [11,12,13,14]. Among these affecting factors, crosslinker is an important factor influencing the properties of RTV silicone rubber because the hydrolysis groups of crosslinker could react with moisture to form Si-OH and then condense with Si-OH of PDMS to form three-dimensional network structure, greatly improving their thermal and mechanical properties [15,16].

Consequently, there are many studies using various kinds of crosslinkers to improve properties of RTV silicone rubber including γ-functional alkoxy silanes, new silanes combining polyhedral oligomeric silsesquioxanes (POSS), dendronized silane polymers and silicon nitrogen polymers [17,18]. However, metal catalysts were needed to enhance cross-linking rate in the preparations of RTV silicone rubber in these studies. The presences of metal catalysts would bring some drawbacks to RTV silicone rubber, such as generating rubber yellowing, adhesive peak for uneven cross-linking reaction and difficultly in packaging products [19,20]. Thus, it is necessary to develop a catalyst-free curing system to prepare RTV silicone rubber.

Dow Corning Corporation reported a new self-crosslinker for the catalyst-free curing system in the preparation of RTV silicone rubber using tri or tetra functional silanes such as R-Si-Q_3_ (R: alkyl group, Q: hydrolysis groups) [21]. Our group used α-functional ketoxime silanes as self-crosslinkers to prepare RTV silicone rubber, which exhibited better thermal and mechanical properties compared to commercial RTV silicon rubber using metal catalyst [22]. However, the thermodynamics parameters of α-amine ketoximesilanes hydrolysis reaction were not elaborated clearly, which will limit the application of this novel RTV silicon rubber in industrial field because screening the best self-crosslinking agents could save time for industrial processing in the preparation of RTV rubber.

Here, we synthesized five kinds of α-functional ketoxime silanes and discussed the impacts of different functional groups on the hydrolysis activities in the preparations of RTV silicon rubber. Our work could provide hydrolysis kinetic data of self-crosslinkers for this catalyst-free curing system, which is essential for its practical use in industry.

## 2. Experimental

### 2.1. Materials

Chloromethyltrichlorosilane (Industrial grade, Huai’an Debang Chemical Co. Ltd., Huai’an, China) were received without further purification. The following reagents were of analytical grade and used as supplied: methylethylketoxime (MOS, Energy Chemical Co. Ltd., Jinan, China), n-hexane, triethylamine, methylethylketoxime, dichloromethane, tetrahydrofuran, diethylamine, acetonitrile (Tianjin Guangcheng Chemical Reagent Co. Ltd., Tianjin, China), polydimethylsiloxane (PDMS, Mw = 23,700, Jinan Hailan Chemical Co. Ltd., Jinan, China), dibutyltindilaurate (DBTDL, Aladdin Reagent Co., Shanghai, China), n-butylamine, di-n-butylamine, methyltri(methylethylketoxime)silane (MOS, Chengdu Xiya Reagent Co. Ltd., Chengdu, China), ethanol(Jinan Hailan Chemical Co. Ltd., Jinan, China) and ultra pure water (18 MΩ·cm^−1^).

### 2.2. Instruments

The Fourier transform infrared spectroscopy (FT-IR) of KBr pellets of the materials were obtained with a Bruker Tensor-27 spectrometer (Bruker International AG, Rheinstetten, Germany) with the range of 400–4000 cm^−1^ at a resolution of 4 cm^−1^. ^1^H NMR was conducted on BrukerAV 300 MHz and BrukerAV 400 MHz Ultra Shield TM magnet spectrometer (Bruker International AG, Rheinstetten, Germany) using CDCl_3_ as a solvent. Tetramethylsilane was selected as an internal standard (δ = 0.00 ppm). Conductivity was measured by Shanghai Lei Ci DDS-307 conductivity meter (Shanghai Electronics Science Instrument Co., Ltd., Shanghai, China) under the given condition. Weight-loss measurement was conducted in a Mettler Toledo SDTA-854 thermogravimetric analyzer (TGA, Mettler Toledo, Greifensee, Switzerland) under a nitrogen atmosphere from 80 °C to 800 °C at a heating rate of 20 °C·min^−1^.

### 2.3. Synthesis of α-Amine Ketoximesilanes

**Scheme 1:**
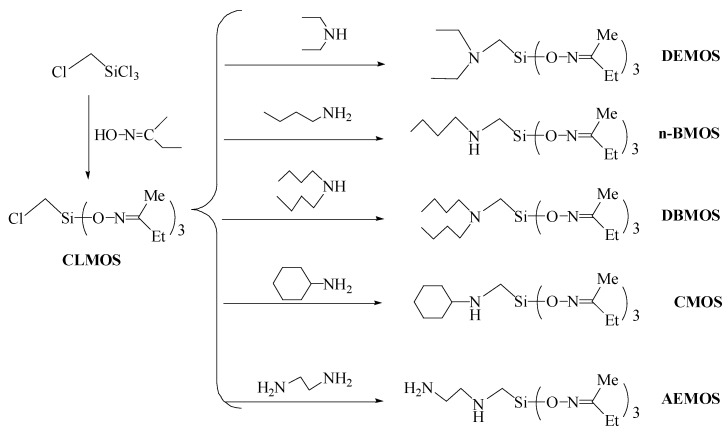
Synthetic routes of α-amine ketoximesilanes.

Scheme 1 illustrates the synthetic routes of α-(N,N-diethyl)aminomethyltri(methylethylketoxime)silane (DEMOS), α-(N,N-di-n-butyl)aminomethyltri(methylethylketoxime)silane (DBMOS), α-(N-n-butyl)aminomethyltri(methylethylketoxime)silane (n-BMOS), α-(N-cyclohexyl)aminomethyltri(methylethylketoxime)silane (CMOS) and α-(β-aminomethyl)aminomethyltri(methylethylketoxime)silane (AEMOS). Chloromethyltri (methylethylketoxime)silane was obtained with methylethylketoxime and n-hexane. In a four-port flask under nitrogen atmosphere, the chloromethyltrichlorosilane (37.06 g) was added into the mixture of methylethylketoxime (105 g) and n-hexane (120 mL) at room temperature under nitrogen atmosphere. After 3 h reaction, the mixture was neutralized with triethylamine and the un-reacted volatile oligomers were removed using reduced pressure distillation. Five kinds of α-amine ketoximesilanes were synthesized using chloromethyltri(methylethylketoxime)silane and five amines (i.e., diethylamine, di-n-butylamine, n-butylamine, cyclohexylamine and β-ethylenediamine). The α-amine ketoximesilanes were added into the mixture of chloromethyltri(methylethylketoxime)silane and acetonitrile with a condensing tube and a stirring system under nitrogen atmosphere. The reaction lasted for 6 h at the temperature of 35 °C. After the reaction, the products were purified by distillation under reduced pressure to remove the unreacted chemicals.

### 2.4. α-Amine Ketoximesilanes Hydrolysis

Five α-amine ketoximesilanes, DEMOS, DBMOS, n-BMOS, CMOS, and AEMOS, were placed in the acidic solution (pH = 6) of water and ethanol (5:13) to investigate the kinetic rates of hydrolysis reaction. The reaction was carried out under the temperature of 25 °C and the humidity of 40%. FI-IR and conductivity measurement were used to record the changes of OH, Si-O-Si stretching vibrations and conductivity to calculate the kinetic rates of α-amine ketoximesilanes, respectively.

In this study, α-amine ketoximesilanes only participate in the hydrolysis reaction via Equation (1) and the rate constant is K. Excess water and ethanol insures this hydrolysis reaction is irreversible. Under the acidic system, the hydrolysis rate is found to be much faster than condensation rates of α-amine ketoximesilanes in the system of water and alcohol. To obtain the hydrolysis rate constant, it was assumed that the hydrolysis completed before the condensation reaction started [23,24]. The equation for expressing the hydrolysis reaction could be described as follows:

d[M]/dt = −K[M](1)

In the above equation, [M] is the molar concentration of α-amineketoximesilanes and K denotes the hydrolysis rate constant. As reported previously, the calibration curves of α-amineketoximesilanes concentration were linearly correlated with the absorption intensity of OH and Si-O-Si measured by FT-IR as well as conductivity, which could be described by the equation of y = kx. In this equation, y denotes concentration of α-amineketoximesilanes [M] and x denotes the absorption intensity or conductivity ([A]). Integration of Equation (1) gives Equation (2):

ln[M]_o_/[M] = ln [A]_o_/[A] = Kt (2)

In Equation (2), t denotes the reaction time. [M]_o_ and [A]_o_ refer to the initial concentration and absorption intensity of α-amineketoximesilanes, respectively. Therefore, the initial slope of the ln [A]_o_/[A] versus time curve can be used to predict the magnitude of hydrolysis rate constant (K). The reaction mixture was transparent and homogenous during the whole hydrolysis process.

### 2.5. Preparation of RTV Silicone Rubber

A series of crosslinkers, i.e., MOS, DEMOS, DBMOS, n-BMOS, CMOS, and AEMOS (0.01–0.1% by weight), was added to the PDMS (Mw = 23,700), respectively, in a double roller mixer. The curing temperature and humidity were maintained at the 25 °C and 40%, respectively, as recommended by China’s standard method for curing RTV silicone rubber (DL/T 627-2012). After mixing for certain time, the simple RTV silicone rubber were prepared for thermal stability measurement. Briefly, a certain amount of silica (SiO_2_) was added with a magnetic stirrer into PDMS, which was placed in a 100 mL beaker. After mixed uniformly, the mixture was placed in an oven at 150 °C for 15 min. After cooling, the mixture was dissolved in n-hexane totally. Then, certain amounts of crosslinkers were added into the solution with intense agitation. Finally, the mixture was cured in a steel mold at required temperature to accomplish curing and obtain the RTV silicone rubber.

## 3. Results and Discussion

### 3.1. Synthesis and Characterization

**Figure 1:**
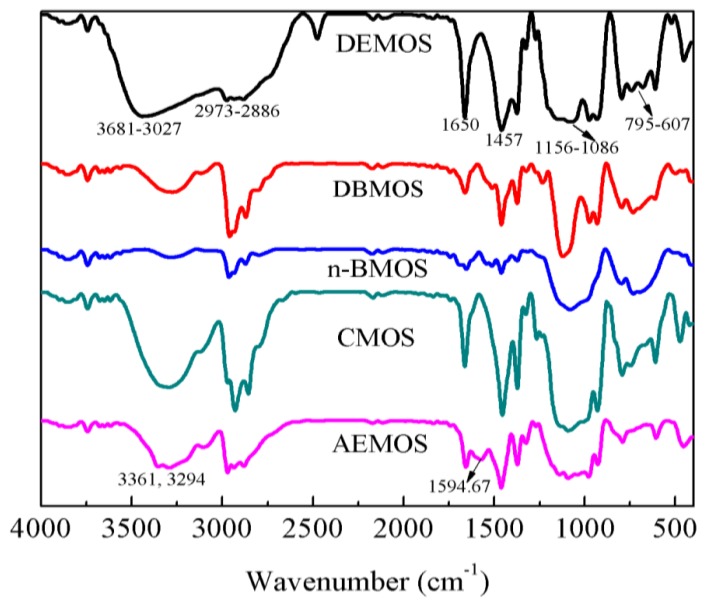
FT-IR spectrums of α-amine ketoximesilanes. DEMOS, α-(N,N-diethyl)aminomethyltri (methylethylketoxime)silane; DBMOS, α-(N,N-di-n-butyl)aminomethyltri(methylethylketoxime)silane; n-BMOS, α-(N-n-butyl)aminomethyltri(methylethylketoxime)silane; CMOS, α-(N-cyclohexyl)aminomethyltri(methylethylketoxime)silane; AEMOS, α-(β-aminomethyl) aminomethyltri(methylethylketoxime)silane.

The five crosslinkers DEMOS, DBMOS, n-BMOS, CMOS, and AEMOS were characterized by FT-IR and ^1^H NMR (Figure 1 and Figures S1–S5). As shown in Figure 1, the strong broad peaks at 1156–1086 cm^−1^ indicated Si-O stretching in Si-ONC groups and the peak at 1274 cm^−1^ was caused by the stretching vibrations of C-N bands. Furthermore, C=N stretching in HO-N=CMeEt induced the moderate peaks at 1650 cm^−1^. The peaks at about 2800 cm^−1^ and around 1400 cm^−1^ were due to C-H stretching and bending vibrations of alkyl groups. All these stretching and bending vibrations of functional groups proved the target compounds were successfully obtained.

Correspondingly, ^1^H NMR resonances (400 MHz, CDCl_3_) arising from the synthesized compounds were described as following: DEMOS, δ 2.31 (q, 6H), 2.14 (q, 4H), 1.95 (s, 1H), 1.82 (s, 9H), 1.00 (t, 9H), 0.98 (t, 6H); DBMOS, δ 2.48 (t, 4H), 2.33–2.08 (q, 6H), 1.82 (s, 9H), 1.72 (s, 2H), 1.43–1.33 (m, 4H), 1.33–1.22 (m, 4H), 1.03–0.91 (t, 9H), 0.87 (t, 6H); n-BMOS, δ 2.81–2.75 (q, 2H), 2.35 (dd, 1H), 2.25–2.12 (q, 6H), 1.97 (s, 9H), 1.82–1.78 (d, 2H), 1.48–1.62 (dd, 1H), 1.35–1.25 (m, 1H), 1.00 (t, 9H), 0.85 (t, 3H); CMOS, δ 2.50 (dd, 1H), 2.33 (q, 6H), 2.18 (m, 1H), 1.82 (d, 2H), 1.71 (s, 9H), 1.67–1.58 (q, 4H), 1.57–1.50 (m, 2H), 1.25–1.06 (m, 4H), 1.01–0.95 (t, 9H); AEMOS, δ 2.78 (q, 2H), 2.40 (m, 2H), 2.26–2.12 (q, 6H), 1.98 (q, 2H), 1.82 (s, 9H), 1.42 (m, 1H), 1.23 (t, 2H), 1.04 (t, 9H) (Figures S1–S5).

### 3.2. Kinetic Rate of α-Amine Ketoximesilanes Hydrolysis

**Figure 2:**
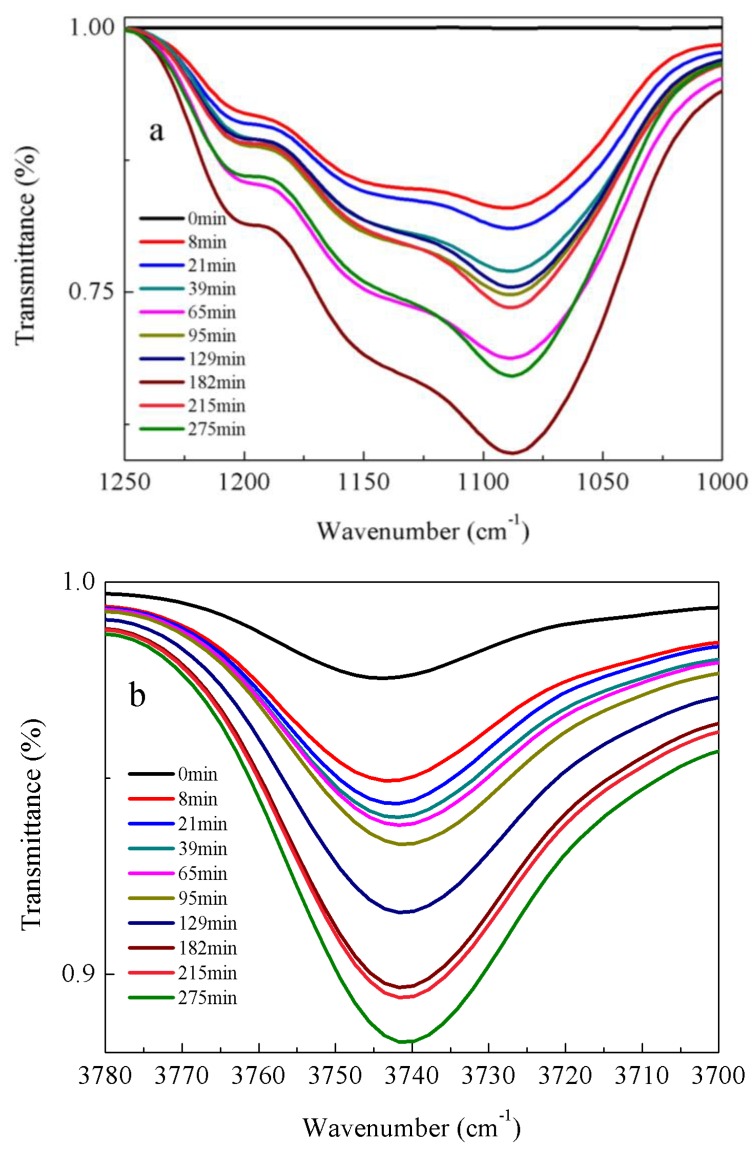
FT-IR spectra of α-(N,N-diethyl)aminomethyltri(methylethylketoxime)silane (DEMOS) in the presences of ethanol and water under different hydrolysis time: (**a**) Si-O-Si stretching vibrations; and (**b**) OH stretching vibrations. The mass ratio of AEMOS, water and ethanol was 1:1:5. The experiments were performed at 25 °C and 40% humidity.

**Figure 3:**
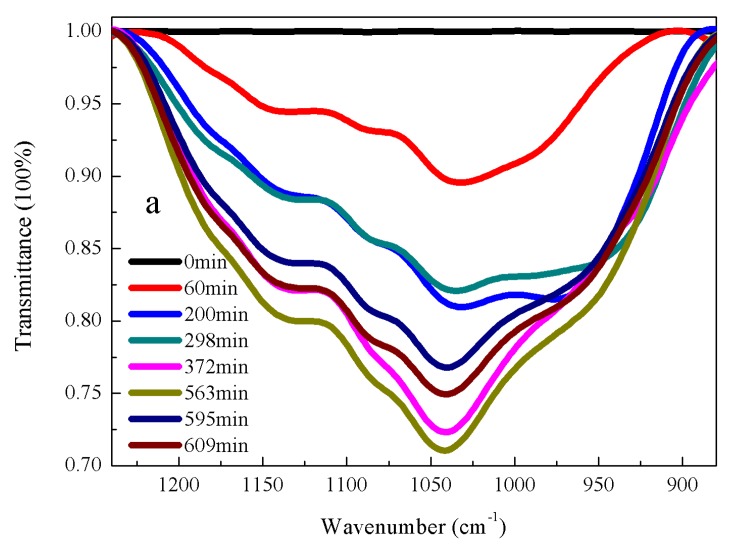

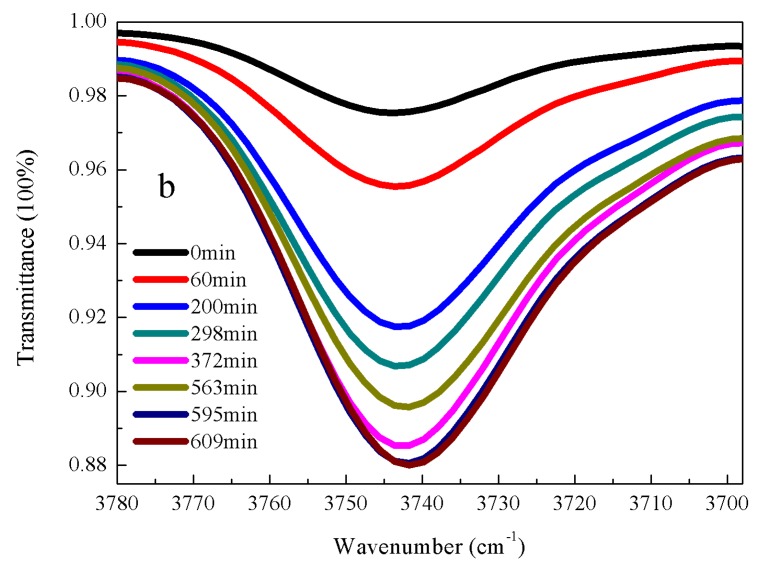
FT-IR spectrums of α-(β-aminomethyl)aminomethyltri(methylethylketoxime)silane (AEMOS) in the presences of ethanol and water under different hydrolysis time: (**a**) Si-O-Si stretching vibrations; and (**b**) OH stretching vibrations. The mass ratio of AEMOS, water and ethanol was 1:1:5. The experiments were performed at 27 °C and 35% humidity.

FT-IR spectroscopy and conductivity are quantitative tools for determining the concentrations of target species in solution. From a theoretical view, the concentration of species should be lined with the signal changes of measurement for a given set of sample parameters. Hence, the quantitative information on the concentration changes of measured species could be obtained using signal changes of measurement from the calibration equation [22]. Figure 2 and Figure 3 show the evolution of FT-IR spectra with time for DEMOS and AEMOS in the hydrolysis system of ethanol and water. The integrated intensity of peak at 1050 and 3740 cm^−1^ were used for quantitative analysis because these peaks were strong and without any overlap. The Si-O-Si band intensity increases as the reaction time continues, suggesting the condensation reaction of hydrolyzed silane takes place simultaneously in the hydrolysis system. Our kinetic data demonstrate the reaction rates of two process: the hydrolysis process and condensation process of hydrolyzed silane. Because the approach using FT-IR spectroscopy and conductivity allows for convenient determinations of silane concentrations for calculating thermodynamics parameters of reactions compared to other free radical monitoring approaches [22], calculating the respective kinetic data of hydrolysis process and condensation process will not significantly improve our understanding of this material in the use of industrial field but necessitate the associated increase in experiment cost. Therefore, FT-IR spectroscopy and conductivity are reasonable approaches for monitoring hydrolysis system in our work.

From the conductivity data, the kinetics of α-amine ketoximesilanes hydrolysis exhibited a typical exponential decay, suggesting that the reaction obey the first-order law (Figure S6). Based on the experiment data and Equation (2), the kinetic rates of hydrolysis for DEMOS, DBMOS, n-BMOS, CMOS, and AEMOS were calculated, as shown in Table 1. The good agreement between the experimental data and fitting lines was observed in all the system with R^2^ > 0.5. From the calculated kinetic rates listed in Table 1, it could be seen that the activity of hydrolysis reaction decreased with the order of DEMOS > n-BMOS > DBMOS > CMOS > AEMOS at the given temperature and humidity. The reaction rate constants across the five α-amine ketoximesilanes varied from 12.2 × 10^−4^ s^−1^ to 7.6 × 10^−4^ s^−1^, reflecting the impacts of different functional groups on α-amine ketoximesilanes hydrolysis are not obvious.

### 3.3. Thermal Stability of RTV Silicone Rubber

**Figure 4:**
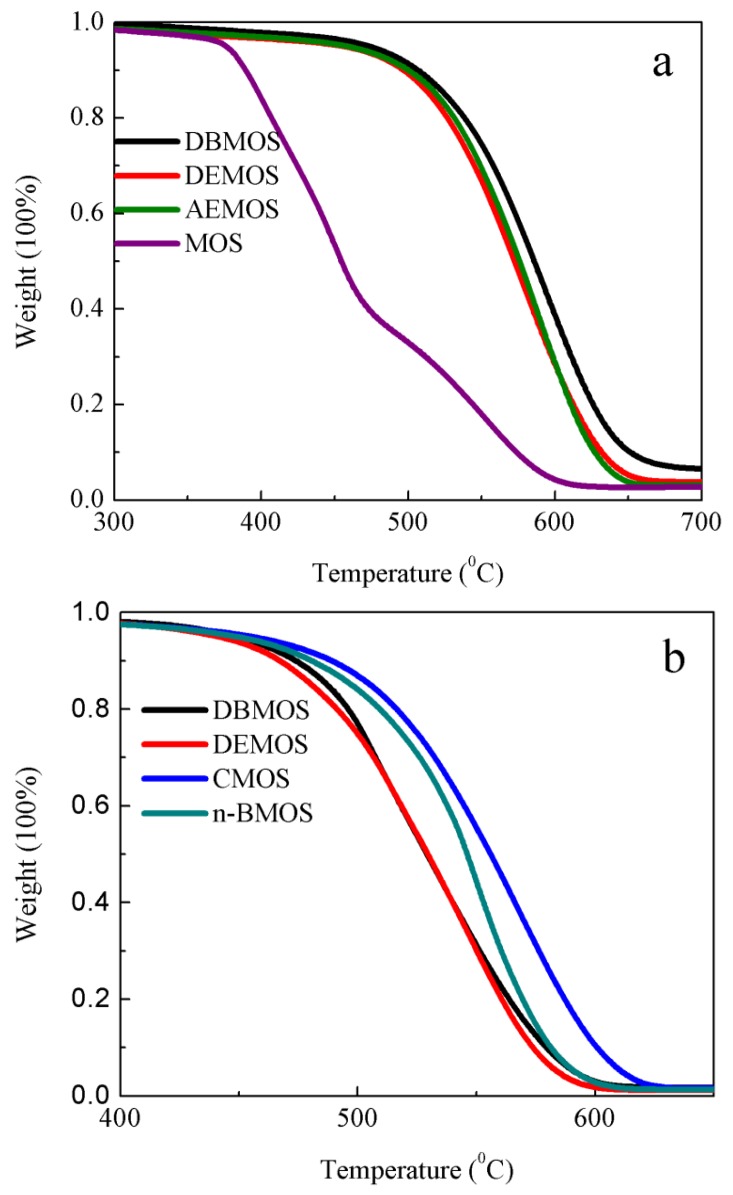
TGA curves of the cross-linked PDMS with different α-amine ketoximesilanes: (**a**) α-amine ketoximesilanes: PDMS = 1:10; and (**b**) α-amine ketoximesilanes: PDMS = 1:45. DEMOS, α-(N,N-diethyl)aminomethyltri(methylethylketoxime)silane; DBMOS, α-(N,N-di-n-butyl)aminomethyltri(methylethylketoxime)silane; n-BMOS, α-(N-n-butyl)aminomethyltri(methylethylketoxime)silane; CMOS, α-(N-cyclohexyl)aminomethyltri(methylethylketoxime)silane; AEMOS, α-(β-aminomethyl) aminomethyltri(methylethylketoxime)silane.

The thermal stabilities of the RTV silicon rubber with α-amine ketoximesilanes and mehtyltri(methylethylketoxime)siliane (MOS) as crosslinkers were evaluated by TGA. As shown in Figure 4 and Figure S7, the degradation temperature (the thermal weight loss consisted 5% of original weight) of pure elastomer and elastomer with MOS as a crosslinker and dibutyltin dilaurate as a catalyst were significantly lower than those with DBMOS, DEMOS and AEMOS as crosslinkers. These results demonstrated that α-amine ketoximesilanes had much more advantage in promoting thermal stability of PDMS compared to the commercial MOS, which is consistent with our previous study [22]. In addition, the degradation temperatures of elastomer with α-amine ketoximesilanes were comparable with each other, indicating different functional groups on α-amine ketoximesilanes had negligible impacts on thermal stability of PDMS.

## 4. Conclusions

A series of α-amine ketoximesilanes were synthesized and applied in the preparations of environment-friendly RTV silicone rubber as self-crosslinkers. FT-IR and ^1^H NMR were used to confirm the structures of α-amine ketoximesilanes. We adopted the FT-IR and conductivity methods to investigate the kinetic process of α-amine ketoximesilanes hydrolysis in the system of water and ethanol. The kinetic constant rates were in the range of 12.2 × 10^−4^ s^−1^ to 7.6 × 10^−4^ s^−1^. The fastest hydrolysis reaction of α-amine ketoximesilanes was DEMOS, followed by n-BMOS, DBMOS, CMOS and AEMOS at the given temperature and humidity. In comparison to RTV silicon rubber using MOS as a crosslinker and dibutyltin dilaurate as a catalyst, the α-amine ketoximesilanes crosslinked RTV silicon rubber exhibited a better performance in thermal stability.

## Figures and Tables

**Table 1:** Reaction rate constants for α-amine ketoximesilanes hydrolysis in the system of ethanol and water (1:5:13) at the temperature of 25 °C and the humidity of 40%.

Compounds	Rate Constant (×10^−4^ s^−1^)	Dependence
DEMOS	12.2	R^2^ = 0.611
n-BMOS	9.1	R^2^ = 0.829
DBMOS	8.6	R^2^ = 0.613
CMOS	8.2	R^2^ = 0.549
AEMOS	7.6	R^2^ = 0.795

DEMOS, α-(N,N-diethyl)aminomethyltri(methylethylketoxime)silane; DBMOS, α-(N,N-di-n-butyl)aminomethyltri(methylethylketoxime)silane; n-BMOS, α-(N-n-butyl)aminomethyl tri(methylethylketoxime)silane; CMOS, α-(N-cyclohexyl)aminomethyltri(methylethylketoxime)silane; AEMOS,α-(β-aminomethyl)aminomethyltri(methylethylketoxime)silane.

## Supplementary Materials

The following are available online at http://www.mdpi.com/1996-1944/11/5/790/s1. Figure S1: The ^1^H NMR spectrum of α-(N,N-diethyl)aminomethyltri(methyleth-ylketoximo)silane (DEMOS), Figure S2: The ^1^H NMR spectrum of α-(N,N-di-n-butyl)aminomethyltri(methylethylketoxime)silane (DBMOS), Figure S3: The ^1^H NMR spectrum of α-(N-n-butyl)aminomethyltri(methylethylketoxime)silane (n-BMOS), Figure S4: The ^1^H NMR spectrum of α-(N-cyclohexyl)aminomethyltri(methylethylketoxime)silane (CMOS), Figure S5: The ^1^H NMR spectrum of α-(β-aminomethyl)aminomethyltri(methylethylketoxime)silane (AEMOS), Figure S6: Conductibility of α-amine ketoximesilanes in the presences of ethanol and water under different hydrolysis time. The mass ratio of α-amine ketoximesilanes, water and ethanol was 1:5:13. The experiments were performed at the temperature of 25 °C and the humidity of 40%, Figure S7: TGA curves of pure PDMS. The degradation temperature (the thermal weight loss consisted 5% of original weight) of pure PDMS was 343 °C. The degradation temperature (the thermal weight loss consisted 5% of original weight) of silicone rubber with CMOS, n-BMOS, DBMOS, DEMOS was 448 °C, 443 °C, 438 °C and 435 °C, respectively.
