# Supplementary material for: Impacts of Different Functional Groups on the Kinetic Rates of α-Amine Ketoximesilanes Hydrolysis in the Preparation of Room Temperature Vulcanized Silicone Rubber
Source: Materials (Basel). 2018 May 13;11(5):790. doi: 10.3390/ma11050790 (PMC5978167; doi:10.3390/ma11050790)
Supplement: Supplementary file 1 [file materials-11-00790-s001.pdf]

Supplementary Materials

Impacts of Different Functional Groups on the Kinetic Rates of α-Amine Ketoximesilanes Hydrolysis in the Preparation of Room Temperature Vulcanized Silicone Rubber

HuihuiXu [1], Zihou Liu [1], Qingyang Liu [2], Yiling Bei [1,*] and Qingzeng Zhu [1]

[1] Key Laboratory of Special Functional Aggregated Materials, Ministry of Education, School of Chemistry and Chemical Engineering, Shandong University, Jinan 250100, China; 201411546@mail.sdu.edu.cn (H.X.); EBLX22@163.com (Z.L.); qzzhu@sdu.edu.cn (Q.Z.)

[2] College of Biology and the Environment, Nanjing Forestry University, Nanjing 210037, China; liuqingyang0807@aliyun.com

* Correspondence: beiyiling@sdu.edu.cn

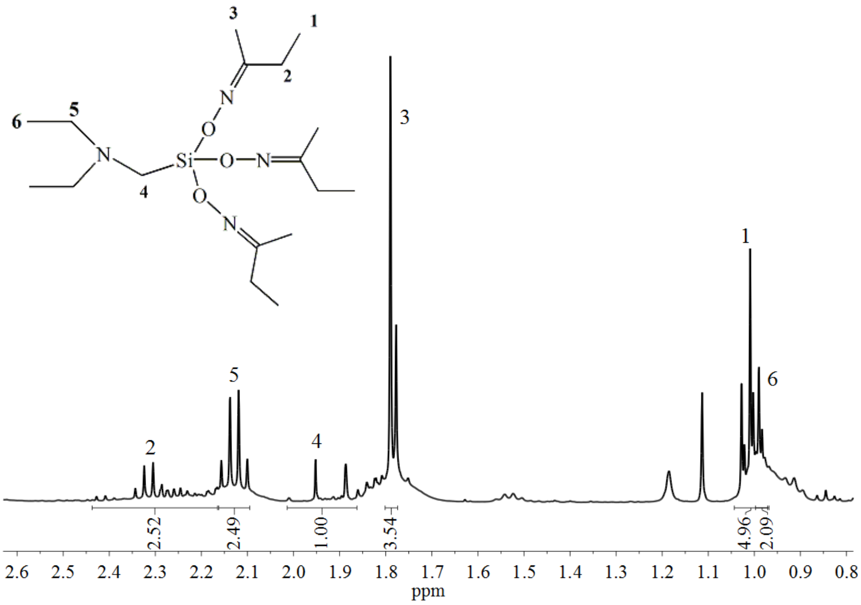

Figure S1. The ^{1}HNMR spectrum of α-(N,N-diethyl)aminomethyltri(methyleth-ylketoximo)silane (DEMOS).

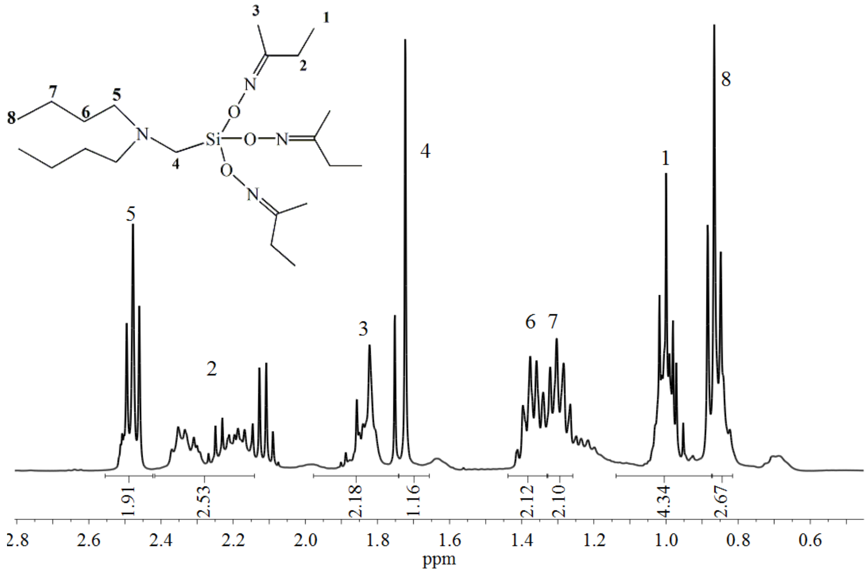

Figure S2. The ^{1}HNMR spectrum of
α-(N,N-di-n-butyl)aminomethyltri(methylethylketoxime)silane (DBMOS).

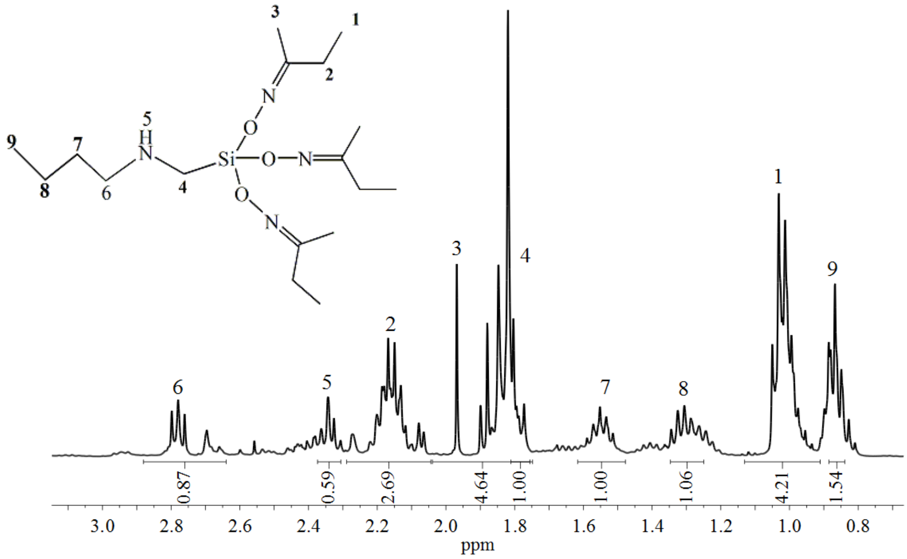

Figure S3. The ^{1}HNMR spectrum of
α-(N-n-butyl)amino methyltri(methylethylketoxime)silane (n-BMOS).

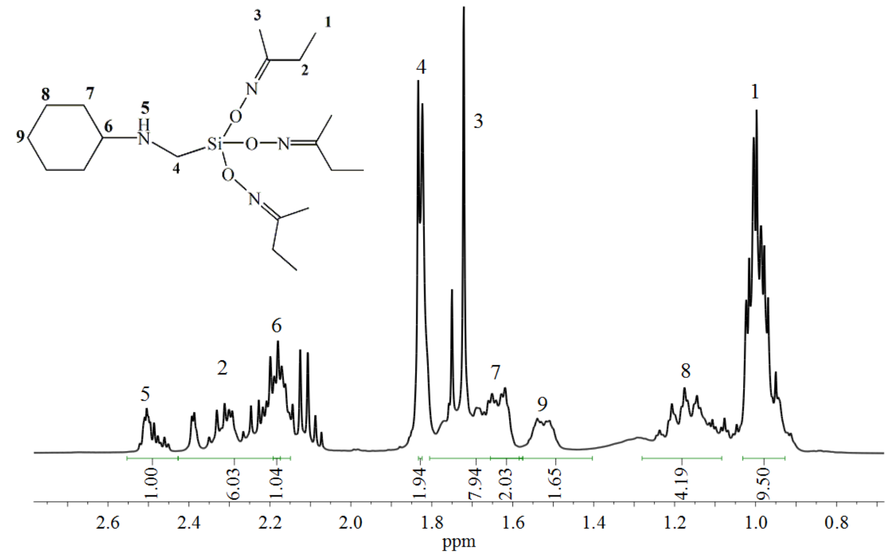

Figure S4. The ^{1}HNMR spectrum of
α-(N-cyclohexyl) aminomethyl tri(methylethylketoxime)silane (CMOS).

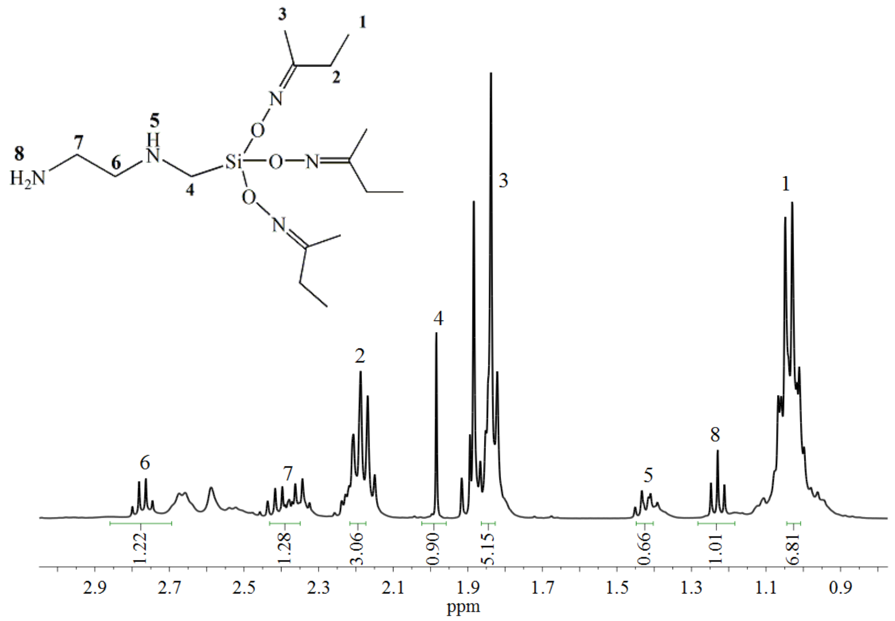

Figure S5. The ^{1}HNMR spectrum of
α-(β-aminomethyl)aminomethyltri(methylethylketoxime)silane (AEMOS).

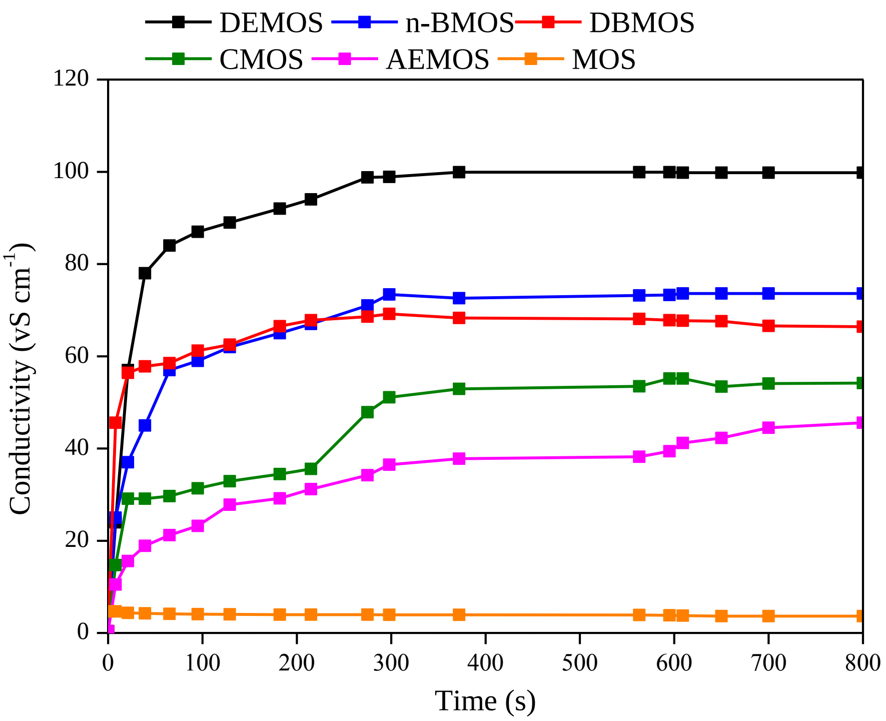

Figure S6. Conductibility of α-amine ketoximesilanes in the presences of ethanol and water under different hydrolysis time. The mass ratio of α-amine ketoximesilanes, water and ethanol was 1:5:13. The experiments were performed at the temperature of 25 °C and the humidity of 40%.

DEMOS, α-(N,N-diethyl)aminomethyltri(methylethylketoxime)silane;

DBMOS, α-(N,N-di-n-butyl)aminomethyltri(methylethylketoxime) silane;

n-BMOS, α-(N-n-butyl)aminomethyl tri(methylethylketoxime) silane;

CMOS, α-(N-cyclohexyl)aminomethyltri(methylethylketoxime)silane;

AEMOS, α-(β-aminomethyl)aminomethyltri(methylethylketoxime)silane.

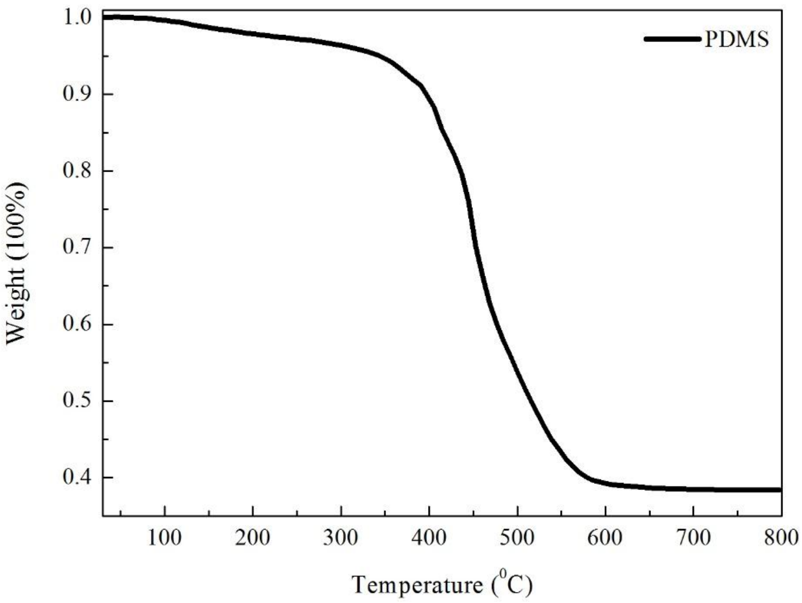

Figure S7. TGA curves of pure PDMS. The degradation temperature (the thermal weight loss consisted 5% of original weight) of pure PDMS was 343 °C. The degradation temperature (the thermal weight loss consisted 5% of original weight) of silicone rubber with CMOS, n-BMOS, DBMOS, DEMOS was 448 °C, 443 °C, 438 °C and 435 °C, respectively.
